# Current status of medical students’ use of large language models for graduation thesis writing: a comparative cross-sectional survey

**DOI:** 10.3389/fmed.2026.1948768

**Published:** 2026-10-12

**Authors:** Xuexiu Liu, Junyang Luo, Yanhan Chen, Jianjun Zhu, Yi Liu, Zhengjie Wang

**Affiliations:** 1 Department of Pediatrics, The First Affiliated Hospital of Chongqing Medical University, Chongqing, China; 2 Department of Nuclear Medicine, The First Affiliated Hospital of Chongqing Medical University, Chongqing, China; 3 College of Nursing, Chongqing Medical University, Chongqing, China; 4 School of Basic Medical Sciences, Shanxi Medical University, Shanxi, China; 5 Department of Nuclear Medicine, Fudan University Shanghai Cancer Center, Shanghai, China

**Keywords:** graduation thesis, large language models, medical students, questionnaire survey, technology acceptance mode

## Abstract

**Objective:**

Guided by the Technology Acceptance Model (TAM) as a conceptual framework, this study explored medical students’ use of large language models (LLMs) in graduation thesis writing, focusing on application patterns, perceptions, and potential risks.

**Methods:**

A comparative cross-sectional questionnaire survey using convenience sampling was conducted among medical graduates from medical schools in China in 2024 and 2025. The self-designed 25-item questionnaire included five-point Likert items on perceived advantages and disadvantages of LLMs. Internal consistency was assessed using Cronbach's *α*, exploratory factor analysis (EFA) was performed, and univariable and multivariable logistic regression analyses were used to identify factors associated with LLMs use.

**Results:**

A total of 341 valid questionnaires were collected. Overall, 54.25% (185/341) of respondents reported using LLMs in graduation thesis writing, with use reported by 27.37% (52/190) in 2024 and 88.08% (133/151) in 2025. ChatGPT was the most frequently used platform in 2024 (82.69%, 43/52), whereas Deepseek was the most frequently used platform in 2025 (88.72%, 118/133). LLMs were used primarily during the literature review stage (74.05%, 137/185), mainly to provide writing suggestions (82.16%, 152/185). Cronbach's *α* was 0.720 for perceived advantages and 0.650 for perceived disadvantages, and exploratory factor analysis identified a two-factor clustering pattern consistent with the predefined grouping of perceived advantages and disadvantages. Among LLMs users, 68.65% (127/185) believed that LLMs use would not compromise academic integrity, whereas 47.57% (88/185) remained concerned about potential academic misconduct. The main perceived disadvantages included the risk of academic misconduct, limited content accuracy, and data privacy and security concerns. The findings were discussed with reference to TAM-related concepts as an interpretive framework.

**Conclusion:**

LLMs use was substantially higher in the 2025 survey than in the 2024 survey. Although LLMs improve writing efficiency and provide valuable writing support, concerns regarding academic integrity, content accuracy, and data privacy remain important considerations. The TAM provides a useful framework for interpreting students’ adoption patterns and may inform the development of AI education and governance strategies in medical education.

## Introduction

With the rapid development of artificial intelligence (AI), AI has gradually penetrated many aspects of medical education and scientific research (1). Among AI technologies, Large Language Models (LLMs), represented by ChatGPT and Deepseek, have become important auxiliary tools for scientific manuscript preparation. Their powerful natural language processing capabilities provide clear advantages in grammar correction, expression polishing, generation of literature-review drafts, and writing inspiration (2). Several authoritative journals have also recognized their potential for improving writing quality and efficiency (3). These models can reduce students' workload, save time, and enhance language quality (4). Unlike journal articles, degree theses are broader in scope and structurally more complex, encompassing literature review, research methods, data analysis, and other components. The technology can support students by generating preliminary drafts, explaining professional concepts, assisting with data analysis, and checking manuscript formatting, thereby improving the overall writing experience (5). However, the application of AI still faces important challenges, including the reliability of generated content, the risk of plagiarism, and concerns regarding data privacy and ethics (6, 7). These challenges constitute important dimensions of users' perceived risk and may substantially influence students' willingness to adopt the technology.

The Technology Acceptance Model (TAM) provides a mature theoretical framework for understanding and predicting users' willingness and behavior when adopting new technologies (8). The core variables of these models, including perceived usefulness, perceived ease of use, social influence, and facilitating conditions, have been widely used to evaluate acceptance of educational technology tools (9). Previous studies have applied TAM-related concepts to explore factors influencing acceptance of LLMs (10, 11). TAM therefore provides a conceptual framework for interpreting the psychological and contextual factors related to medical students' use of LLMs in graduation thesis writing.

Guided by the TAM as a conceptual framework, this study investigated medical graduates' use of LLMs in degree-thesis writing, explored their user experience and perceptions, and examined the current application status, attitudes, and potential barriers to technology adoption. Through empirical analysis, the study identifies existing challenges in the application of the technology and proposes recommendations for educational practice and institutional governance, with the aim of promoting the responsible, standardized, and effective use of AI in medical education.

## Methods

### General information

This comparative cross-sectional study used convenience sampling to survey medical graduates in China in 2024 and 2025. The inclusion criteria were as follows: (1) professional background: medical students from medical colleges and universities in various provinces, municipalities, and autonomous regions of China; (2) technical experience: students could participate regardless of whether they had experience using LLMs; and (3) informed consent: participants voluntarily participated in the study and agreed to complete the questionnaire. The exclusion criteria were as follows: (1) incomplete questionnaires or missing key variables; (2) obvious logical errors or contradictory responses; and (3) duplicate submissions, for which only the first valid questionnaire was retained. Questionnaires with incomplete data were excluded from data analysis as appropriate. This study was approved by the Ethics Committee of the Children's Hospital of Chongqing Medical University (approval No.: 2024-263).

### Survey method

A self-designed questionnaire was used in this study. The first version of the questionnaire was developed based on a literature review and expert consultation, and was subsequently discussed and revised several times by the research team. Before the formal survey, 20 medical graduate students were selected for a pilot test. Based on their feedback, the questionnaire was further revised through expert-group discussion. An expert meeting was then held, inviting six experts in medicine and education (all with associate-professor or higher professional titles, master's degrees or above, and at least 10 years of work experience) to provide opinions. Several items were adjusted according to their suggestions to improve the relevance and clarity of the questionnaire. After revision, the questionnaire was again submitted to the experts for confirmation of content validity. Through this iterative revision process, the final formal questionnaire contained 25 items.

The questionnaire consisted of three parts. The first part collected basic information, including sex, age, specialty, degree type, academic level, primary data sources for thesis research, and weekly time spent on thesis writing. The second part assessed medical graduate students' use of LLMs during thesis writing, including whether they used the technology, the specific tools adopted, the main stages of application, weekly time spent using these tools, their primary functions in the writing process, satisfaction with the generated content, perceptions of their impact on academic integrity, concerns about potential academic-integrity issues, perceived effects on thesis quality, motivations for use, challenges encountered, the sections of the thesis to which they contributed most, methods used to revise AI-generated content, willingness to recommend them to others, the proportion of the thesis completed with their assistance, the perceived proportion of classmates using them, reasons for nonuse, and future willingness to adopt the technology. The third part assessed respondents' perceptions of LLMs, including their perceived advantages and disadvantages, whether medical schools should provide training on their use, views on their future application in academic research, and evaluations of their potential in medical-paper writing. Perceived advantages and disadvantages were each assessed using five items rated on a five-point Likert scale (1 = strongly disagree, 5 = strongly agree). These 10 Likert-scale items were completed by all respondents, including both LLMs users and non-users. The questionnaire link was https://www.wjx.cn/newwjx/manage/myquestionnaires.aspx?randomt=1719716802. Content validity was evaluated by the same six-member expert panel, comprising three physicians and three teachers. The content validity index of the questionnaire was 0.93. Because the questionnaire was designed to explore the current status and perceptions of LLMs use rather than to validate a theoretical model, standardized TAM scales were not included.

### Data collection

A one-to-one standardized guidance approach was adopted. Students were informed about the purpose, content, target population, and inclusion criteria of the survey, and the Wenjuanxing link was distributed to invite eligible participants to complete the questionnaire. Respondents completed the questionnaire independently according to their actual situation. Quality-control measures included: (1) use of an anonymous questionnaire; (2) IP restrictions so that each respondent could answer only once; (3) mandatory-answer logic, preventing submission when items were missing; and (4) manual review and removal of invalid questionnaires with obvious regular patterns of response. All data were entered independently by two researchers. Any discrepancies were checked jointly by both researchers, and a third researcher performed the final review of all data.

### Statistics

Data collected through Wenjuanxing were imported into Excel for double-person verification and then imported into R 4.5.2 for statistical analysis. Quantitative variables were first tested for normality. Normally distributed data were expressed as mean +/- standard deviation. Five-point Likert items were summarized using mean and standard deviation, an approach supported by evidence on the robustness of parametric summaries for Likert-scale data (12). Qualitative data were described using frequencies, composition ratios, and percentages. Comparisons between two groups were performed using the Pearson chi-square test, and Fisher's exact test. Internal consistency of the five perceived-advantages items and five perceived-disadvantages items was assessed using Cronbach's *α* (13). Sampling adequacy for factor analysis was evaluated using the Kaiser-Meyer-Olkin measure and Bartlett's test of sphericity. Parallel analysis was used to determine the number of factors, and EFA was performed using a polychoric correlation matrix, principal-axis factoring, and oblimin rotation (14). Univariable and multivariable binary logistic regression analyses were used to identify factors associated with LLMs use, with survey year, sex, age, specialty, degree type, and academic level included in the multivariable model irrespective of their significance in the univariable analyses. Age was grouped as ≤25, 26–30, and ≥31 years, and specialty was grouped as clinical medicine, nursing, and other specialties. Odds ratios (ORs) and adjusted ORs with 95% confidence intervals (CIs) were reported. All statistical tests were two-sided, and *P* < 0.05 was considered statistically significant.

## Results

### Baseline characteristics

A total of 190 valid questionnaires were collected in 2024 and 151 in 2025. Most participants were aged 26–30 years, accounting for 46.04% (157/341). Master's students accounted for 90.91% (310/341), and professional-degree graduate students accounted for 68.33% (233/341). The main specialties were clinical medicine and nursing, accounting for 40.47% (138/341) and 49.85% (170/341), respectively. Detailed demographic information is shown in Table 1.

**Table 1:** Basic information of respondents.

Item	2024 *n* = 190 (55.72%)	2025 *n* = 151 (44.28%)	Total *n* (%)
Gender			
Male	38 (20.00)	24 (15.89)	62 (18.18)
Female	152 (80.00)	127 (84.11)	279 (81.81)
Age (years)			
<20	1 (0.50)	0 (0)	1 (0.29)
20–25	64 (33.70)	79 (52.32)	143 (41.94)
26–30	119 (62.60)	38 (25.17)	157 (46.04)
31–35	4 (2.10)	18 (11.91)	22 (6.45)
≧36	2 (1.10)	16 (10.60)	18 (5.28)
Degree type			
Professional degree	131 (68.90)	102 (67.55)	233 (68.33)
Academic degree	59 (31.10)	49 (32.45)	108 (31.67)
Academic level			
Master's student	173 (91.10)	137 (90.73)	310 (90.91)
Doctoral student	17 (8.90)	14 (9.27)	31 (9.09)
Specialty			
Clinical medicine	94 (49.47)	44 (29.14)	138 (40.47)
Basic medicine	15 (7.89)	3 (1.99)	18 (5.28)
Public health	1 (0.53)	0 (0)	1 (0.29)
Pharmacy	5 (2.63)	0 (0)	5 (1.47)
Nursing	72 (37.89)	98 (64.90)	170 (49.85)
Other	3 (1.58)	6 (3.97)	9 (2.64)

### Current status of medical students' use of LLMs in graduation thesis writing

Among all participants, 54.25% (185/341) used LLMs in graduation thesis writing. The use rate was 27.37% (52/190) in 2024 and 88.08% (133/151) in 2025. In 2024, ChatGPT had the highest use rate (82.69%, 43/52); whereas Deepseek became the most frequently used platform in 2025 (88.72%, 118/133). The primary application of these tools was to provide writing suggestions (82.16%, 152/185). Among the 341 respondents, 185 reported using them, and 74.05% (137/185) primarily applied them during the literature review stage. Details are shown in Figure 1. Among users, 47.03% (87/185) believed that the technology contributed to 10%–30% of their thesis writing. Compared with the overall weekly time spent on thesis writing, respondents devoted less time to AI-assisted writing, with 61.08% (113/185) spending <5 h per week using these tools, whereas the overall time spent on thesis writing was predominantly 5–10 h per week (Figure 2).

**Figure 1:**
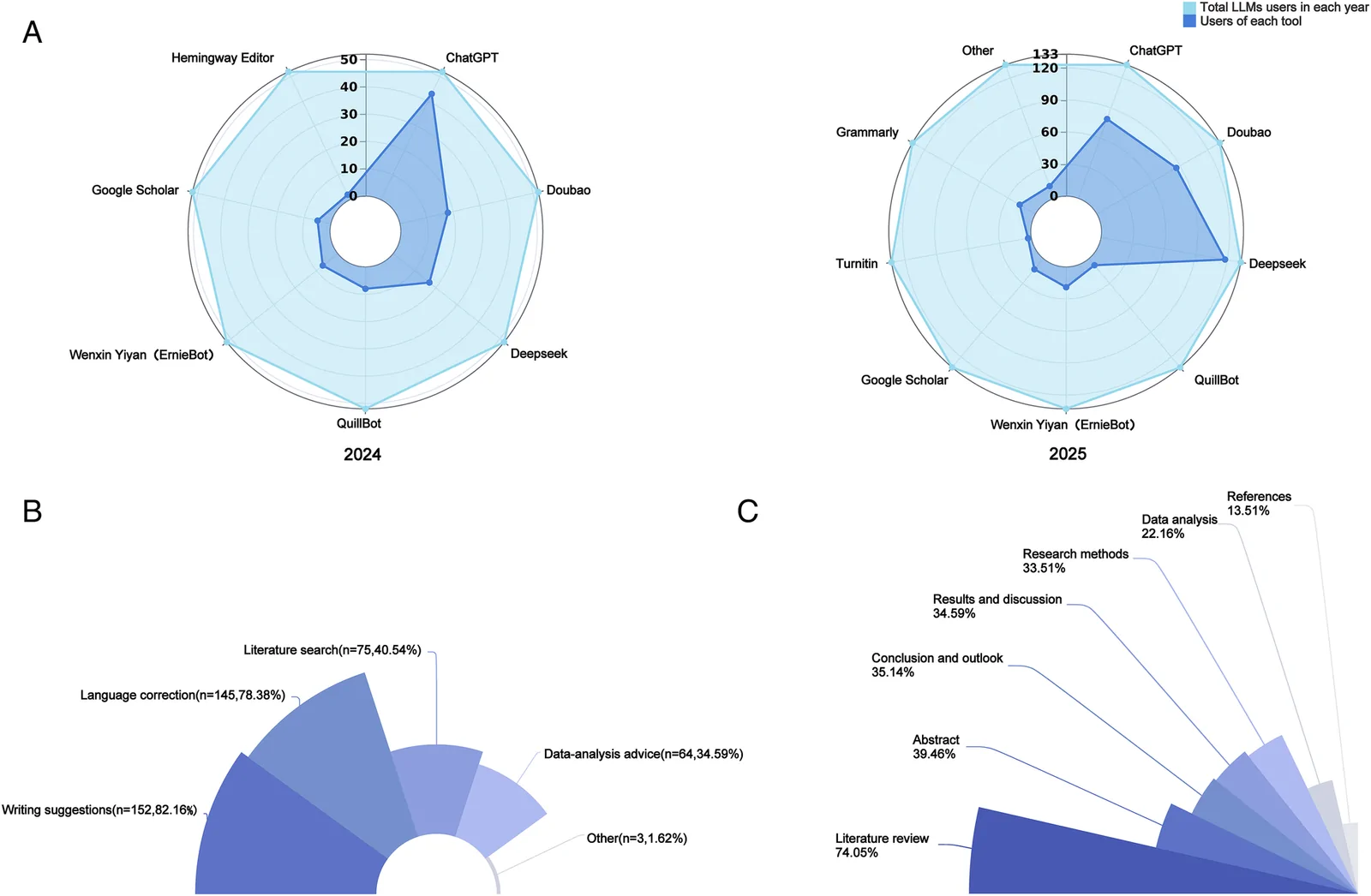
Distribution of tools, purposes, and stages of LLMs use among medical students during thesis writing. **(A)** Tools used in the 2024 and 2025 survey cohorts. **(B)** Purposes of LLMs use. **(C)** Thesis sections or stages supported by LLMs. Multiple responses were permitted.

**Figure 2:**
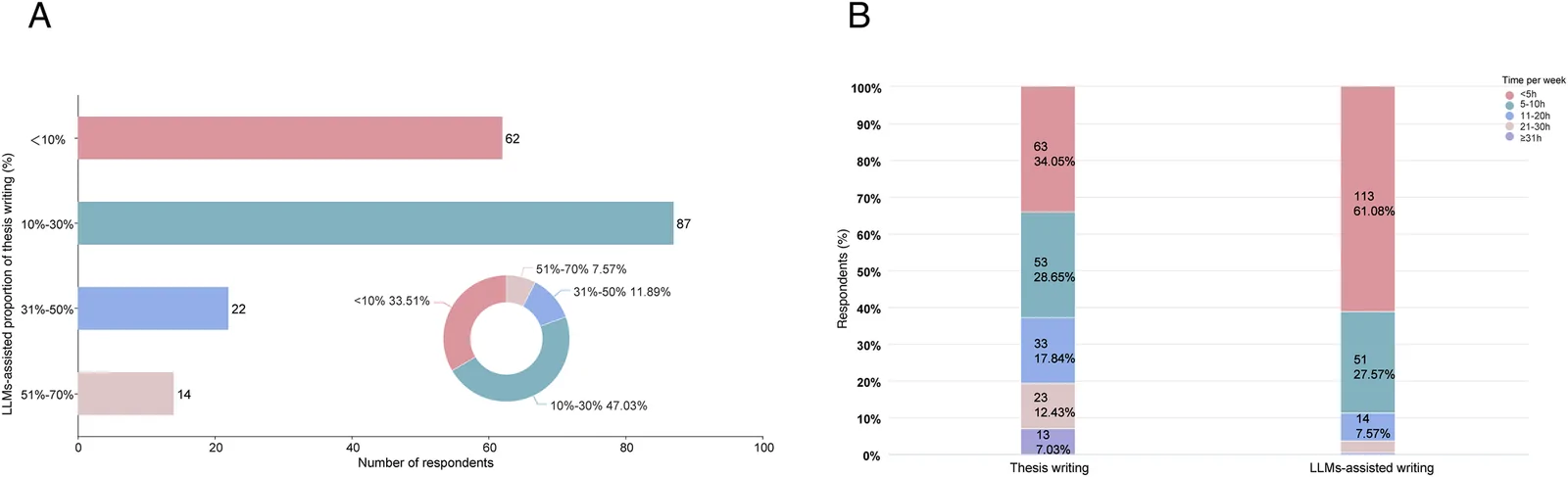
Reported contribution of LLMs to thesis writing and comparison of weekly writing time. **(A)** Proportion of thesis writing assisted by LLMs. **(B)** Weekly time spent on thesis writing and LLMs-assisted writing.

### Medical students' perceptions of using LLMs in thesis writing

Among LLMs users, 68.65% (127/185) believed that using LLMs would not compromise academic integrity, whereas 47.57% (88/185) remained concerned about potential academic misconduct. Cross-tabulation analysis further revealed that among respondents who perceived no negative impact of LLMs use on academic integrity, 41.1% (52/127) still expressed concerns about potential academic misconduct (Supplementary Table S2). These findings indicate that students may simultaneously recognize the utility of LLMs while remaining cautious about their potential misuse. These findings indicate that, regardless of adoption status, academic-integrity concerns remained the primary barrier to the use of generative AI in graduation thesis writing.

### Advantages and disadvantages of using LLMs

All items related to the advantages and disadvantages of LLMs were scored using a five-point Likert scale (1 = strongly disagree, 5 = strongly agree). After data collection, each item was described using the mean and standard deviation. The scores for the advantages and disadvantages of LLMs use showed that, in 2024, the highest-scoring advantages were improvement of language expression and correction of grammar and spelling errors, indicating the most prominent perceived benefits. This finding was consistent with the main uses of LLMs in the questionnaire. The highest-scoring disadvantage was potential academic misconduct, such as plagiarism or overreliance, consistent with the concern about academic-integrity effects reported by 47.57% of respondents. In contrast, the item “privacy and security risks, such as data leakage” had the lowest score, suggesting that this issue had not yet been widely regarded as a major risk in the current usage context.

The 2025 results showed that the score for the advantage “providing accurate data-analysis methods and suggestions” increased compared with 2024, suggesting that respondents increasingly recognized the value of these models in supporting research methodology. At the same time, among the disadvantages, the item “inaccurate content generation, which may lead to misleading information” had the lowest score, reflecting greater user trust in the accuracy of AI-generated output and suggesting that the performance of the technology has continued to improve (Table 2).

**Table 2:** Perceived advantages and disadvantages of LLMs use in the 2024 and 2025 survey cohorts.

Item	2024 score *n* = 190	2025 score *n* = 151	t	*P*
Advantages				
Optimizes language expression and corrects grammar and spelling errors	4.65 ± 0.68	4.16 ± 0.78	6.095	<0.001
Improves writing efficiency and can generate large amounts of text in a short time	4.42 ± 0.90	4.15 ± 0.78	2.965	0.003
Provides inspiration and creativity and stimulates new directions for thinking	4.26 ± 0.88	4.03 ± 0.79	2.539	0.012
Assists literature searches and improves the comprehensiveness of literature reviews	4.17 ± 0.95	3.72 ± 0.54	5.505	<0.001
Provides accurate data-analysis methods and suggestions	3.87 ± 0.92	3.95 ± 0.85	−0.832	0.406
Disadvantages				
May lead to academic misconduct, such as plagiarism or overreliance	4.08 ± 0.79	3.97 ± 0.76	1.305	0.193
Generated content may be inaccurate and may lead to misinformation	3.88 ± 0.89	3.77 ± 0.87	1.148	0.252
May limit original thinking and affect independent thinking ability	3.65 ± 0.82	3.78 ± 0.82	−1.454	0.147
Generated content may lack depth and professionalism	3.63 ± 0.81	3.79 ± 0.85	−1.763	0.079
Security and privacy issues, including the risk of data leakage	3.55 ± 1.03	3.96 ± 0.81	−4.115	<0.001

### Reliability and exploratory factor analysis

Psychometric analysis was performed for the 10 Likert-scale items assessing perceived advantages and disadvantages of LLMs use. Cronbach's *α* was 0.720 for the five perceived-advantages items and 0.650 for the five perceived-disadvantages items. The Kaiser-Meyer-Olkin measure was 0.749, and Bartlett's test of sphericity was significant (*χ*^2^ = 867.68, df = 45, *P* < 0.001). Parallel analysis supported a two-factor solution. The five perceived-advantages items loaded primarily on the first factor (0.554–0.736), whereas the five perceived-disadvantages items loaded primarily on the second factor (0.396–0.714), with minimal cross-loadings (Supplementary Table S1).

### Univariable and multivariable analysis of factors associated with LLMs use

Univariable and multivariable logistic regression analyses are shown in Table 3. In the multivariable model, compared with respondents surveyed in 2024, respondents in the 2025 survey sample showed higher odds of reporting LLMs use after adjustment for demographic characteristics. Notably, these two groups represent separate convenience samples, so this estimate reflects cross-sample differences rather than a temporal causal effect. Compared with the 2024 sample after adjustment for sex, age, specialty, degree type, and academic level (adjusted OR = 21.29, 95% CI 10.70–42.34; *P* < 0.001).

**Table 3:** Univariable and multivariable logistic regression analyses of factors associated with LLMs use.

Variable	Unadjusted OR (95% CI)	*P* value	Adjusted OR (95% CI)	*P* value
Survey cohort				
2024	Reference	–	Reference	–
2025	19.61 (10.91–35.25)	<0.001	21.29 (10.70–42.34)	<0.001
Sex				
Female	Reference	–	Reference	–
Male	1.42 (0.81–2.50)	0.220	3.26 (1.54–6.91)	0.002
Age				
≤25 years	Reference	–	Reference	–
26–30 years	0.37 (0.23–0.59)	<0.001	0.79 (0.42–1.48)	0.459
≥31 years	1.45 (0.67–3.13)	0.350	0.41 (0.14–1.21)	0.106
Specialty				
Clinical medicine	Reference	–	Reference	–
Nursing	2.44 (1.54–3.87)	<0.001	2.13 (1.08–4.19)	0.029
Other	0.72 (0.33–1.58)	0.415	0.55 (0.20–1.54)	0.256
Degree type				
Professional degree	Reference	–	Reference	–
Academic degree	1.14 (0.72–1.81)	0.574	1.80 (0.91–3.58)	0.093
Academic level				
Master's	Reference	–	Reference	–
Doctoral	0.77 (0.37–1.62)	0.493	0.70 (0.26–1.91)	0.484

Unadjusted ORs were estimated using univariable logistic regression. Adjusted ORs were estimated using multivariable logistic regression controlling for survey cohort, sex, age, specialty, degree type, and academic level; all prespecified covariates were retained irrespective of univariable significance. Age and specialty categories were grouped because of small category sizes. Parentheses show 95% CIs. ORs above or below 1 indicate higher or lower odds of reported LLM use relative to the reference category, respectively. OR, odds ratio; CI, confidence interval.

## Discussion

This study found that 54.25% of respondents reported using LLMs during graduation thesis writing; the usage rate was 27.37% (52/190) in 2024 and 88.08% (133/151) in 2025. ChatGPT was the most commonly used platform in 2024 (82.69%, 43/52), while DeepSeek was the dominant platform in 2025 (88.72%, 118/133). LLMs were primarily applied in the literature review stage (74.05%, 137/185), with the core purpose of obtaining writing suggestions (82.16%, 152/185). Among LLMs users, 68.65% (127/185) believed that using LLMs would not compromise academic integrity, whereas 47.57% (88/185) remained concerned about potential academic misconduct. TAM was adopted solely as a theoretical framework to interpret the findings. The main perceived drawbacks included risks of academic misconduct, inadequate content accuracy, and concerns regarding data privacy and security.

Concerns about academic integrity remained a primary barrier to adoption. Among LLM users, 47.57% remained concerned about the potential impact of the technology on academic integrity, and among nonusers such concerns were the most frequently cited reason for non-adoption. LLMs trained on massive published literature may occasionally produce content highly similar to existing studies, leading to quasi-plagiarism (15–18). Evidence from 《Nature》indicates that AI tools facilitate implicit plagiarism while complicating detection (19, 20). Academic consensus confirms that AI cannot be granted authorship, as it cannot assume scholarly accountability; thus, human authors retain full responsibility for all generated content, and AI utilization must be transparently reported in appropriate manuscript sections (21–24). Such ambiguous academic norms substantially increase users' perceived risk and inhibit adoption intention. From the TAM perspective, elevated perceived risk attenuates the positive effect of perceived usefulness. Consistent with previous evidence, academic concerns do not entirely prevent continuous AI adoption, as user attitudes and subjective norms often override perceived barriers, which aligns with the coexistence of prevalent concerns and ongoing LLMs use observed in this study (25–28).

LLMs-generated summaries are less reliable than human-written ones (29–32). These models may produce flawed reasoning, inaccurate statements and fabricated references, known as “hallucination”, especially when filling knowledge gaps (33–37). Such limitations of ChatGPT have attracted criticism in clinical decision-making and academic writing (38–40). Insufficient understanding of model mechanisms partly accounts for this issue; targeted training on model strengths and limitations supports responsible academic use (41–43). Cross-validation is recommended to improve the credibility of AI outputs (44). Content unreliability impairs perceived usefulness, a core construct in our TAM-based interpretation. Substantial manual checking and revision offset efficiency gains and reduce sustained willingness to use LLMs.

Uploading personal and research data to external AI systems raises broad privacy and security concerns and discourages adoption (45–47). Medical research involves sensitive information including patient records and researchers' unpublished intellectual ideas. Accidental leakage may compromise intellectual property and academic innovation, yet these hazards remain underrecognized by academic institutions. As generative AI permeates medical scholarly writing, data privacy and intellectual property protection should be prioritized in medical students' academic integrity education, consistent with guidance from the National Medical Examination Center. Within the TAM framework, inadequate secure deployment and privacy policies create unfavorable facilitating conditions, raise perceived risk and hinder adoption.

A key finding was the coexistence of perceived benefits and academic integrity concerns among LLMs users. While 68.65% (127/185) of users believed LLMs use would not undermine academic integrity, cross-tabulation revealed that 41.1% (52/127) of these respondents still worried about potential academic misconduct. This inconsistency indicates students do not consider LLMs inherently harmful, but acknowledge risks from inappropriate use such as over-reliance or unreported AI assistance. Educational strategies should enhance students' understanding of LLMs functions, encourage responsible use, and formulate clear institutional guidelines. Within the TAM framework, such concerns represent a major barrier to adoption. Heavy reliance on LLMs for language optimization suggests medical students mostly treat LLMs as sophisticated text-editing tools rather than collaborators for research design and scientific reasoning. This may stem from limited awareness of the models' full capabilities or low perceived usefulness for deeper research work.

The main advantages of LLMs include providing writing suggestions, improving language expression, improving writing efficiency, and providing inspiration. These advantages are particularly helpful for nonnative English-speaking students writing English medical papers, as the technology can help optimize the expression of professional terminology and standardize manuscript sentence patterns, matching the rigor and standardization required in medical writing (48, 49). Most respondents indicated that these tools were used more for detailed polishing during thesis writing and did not replace students' independent thinking. Research has shown that ChatGPT contributes to academic writing in three main ways: providing writing inspiration, improving language and grammar, and assisting literature review (50, 51). ChatGPT can also help researchers generate more creative ideas (52). These advantages are the core drivers of user adoption of LLMs-perceived usefulness. The technology meet users' core needs by lowering writing difficulty, especially language barriers, and improving efficiency. Meanwhile, their intuitive interactive mode also improves perceived ease of use. However, the finding that most respondents relied on AI for no more than 10% of their thesis writing suggests that its perceived usefulness is currently limited to auxiliary functions, and its broader potential to support scientific research has not yet been fully recognized or trusted.

(2) Most respondents believed that LLMs have considerable potential for medical-paper writing (80.64%, 275/341), and 83.87% (286/341) expressed a desire to receive formal training in their use. Such training should cover the basic principles of the technology, practical application skills, privacy protection, and strategies for maintaining academic integrity (53–57). Academic communities and educational institutions should establish clear standards and develop practical guidelines for the responsible use of AI writing tools. In addition, more standardized regulations governing the application of generative AI in academic writing are needed (58). From this TAM interpretive perspective, future optimization strategies should focus on improving key variables that affect users' behavioral intention: (1) improve perceived usefulness and perceived ease of use: Through systematic training, students should be helped to master efficient and precise prompt-engineering skills and to explore the potential of LLMs in deeper scientific-research processes, such as study design and data interpretation, rather than limiting their use to language polishing.reduce perceived risk: Clear and operable academic-use norms should be formulated to clarify boundaries of use, disclosure requirements, and responsibility attribution. Reliable LLMs detection tools should be developed and promoted, and new mechanisms for reviewing academic misconduct should be established. (3) optimize facilitating conditions: Medical schools should provide secure and ethically compliant LLMs writing-assistance tools to protect students' data privacy and academic achievements from the technical source. The compliant use of it should be incorporated into academic-integrity and research-education systems.

### Limitations

This study has several limitations. First, convenience sampling was used, and although participants were recruited from different regions of China, the sample may not fully represent all Chinese medical graduate students, which may limit the generalizability of the findings. The sample was predominantly composed of female participants, master's students, and students majoring in nursing or clinical medicine. The relatively high proportion of nursing students may be related to the accessibility of survey distribution channels rather than reflecting the actual distribution of medical students in China. Because the number of eligible students invited and the distribution of respondents across institutions were not systematically recorded, the representativeness of the sample and the observed association between nursing specialty and LLMs use should be interpreted cautiously. The number of eligible students who received the survey invitation and the institutional distribution of respondents were not systematically recorded, so a conventional response rate and institution-level representativeness could not be assessed. Second, the study primarily relied on self-reported questionnaire data, which are subject to recall bias and social-desirability bias. Third, the psychometric analysis was exploratory. Therefore, TAM-related interpretations should be considered exploratory rather than confirmatory. In addition, this study compared two independent cross-sectional surveys conducted in 2024 and 2025. Therefore, the observed differences reflect population-level differences between the two survey samples. Therefore, causal inference regarding temporal trends cannot be established. Future multicenter longitudinal studies with nationally representative samples and validated TAM-based instruments are warranted to further investigate the determinants and long-term impact of LLMs use in medical education.

## Conclusions

LLMs usage for graduation thesis writing differed markedly between the 2024 and 2025 cross-sectional samples of medical students. Medical students primarily adopted the technology to improve writing efficiency, language expression, and literature organization, while concerns regarding academic integrity, hallucinations, and data privacy remained the major barriers to wider adoption. From this TAM interpretive perspective, these adoption patterns can be interpreted in relation to perceived usefulness, perceived ease of use, perceived risk, and facilitating conditions. To promote the safe and effective use of LLMs, medical schools should establish standardized usage guidelines, strengthen literacy and academic-integrity education, provide secure and compliant platforms, and improve institutional support for responsible AI use. These measures may help maximize the educational benefits of the technology while minimizing potential academic and ethical risks.

## Data Availability

The original contributions presented in the study are included in the article/Supplementary Material, further inquiries can be directed to the corresponding author.
