# Supplementary material for: Current status of medical students’ use of large language models for graduation thesis writing: a comparative cross-sectional survey
Source: Front Med (Lausanne). 2026 Oct 12;13:1948768. doi: 10.3389/fmed.2026.1948768 (PMC13696298; doi:10.3389/fmed.2026.1948768)
Supplement: Supplementary file 1 [file Table1.pdf]

SUPPLEMENTARY TABLE S1. Internal consistency and exploratory factor analysis of questionnaire items

A. Internal consistency and suitability for factor analysis

**Psychometric measure**	**Result**
Cronbach's α, perceived advantages (5 items)	0.720
Cronbach's α, perceived disadvantages (5 items)	0.650
Kaiser-Meyer-Olkin (KMO) measure	0.749
Bartlett's test of sphericity	$\chi^2 = 867.68$, df = 45, $P < 0.001$
Number of factors retained by parallel analysis	2

B. Item-level reliability and exploratory factor analysis

**Questionnaire item**	**Corrected item-total correlation**	**Cronbach's α if item deleted**	**Factor 1: Advantages**	**Factor 2: Disadvantages**
**Perceived advantages**				
Improves writing efficiency	0.426	0.693	0.628	−0.015
Improves language expression	0.490	0.672	0.702	−0.002
Provides inspiration and creativity	0.499	0.665	0.665	0.036
Provides data-analysis methods and suggestions	0.562	0.637	0.736	−0.047
Assists literature searching	0.427	0.695	0.554	0.076
**Perceived disadvantages**				
Generated content may be inaccurate	0.370	0.612	0.069	0.484
May lead to academic misconduct	0.322	0.634	0.187	0.396
May limit original thinking	0.443	0.578	−0.044	0.610
May lack depth and professionalism	0.482	0.555	−0.021	0.714
Privacy and security risks	0.399	0.599	0.008	0.514

Note: Psychometric analyses included all 341 respondents. Corrected item-total correlations and Cronbach's α if item deleted were calculated within the corresponding five-item domain. EFA used a polychoric correlation matrix, principal-axis factoring, and oblimin rotation; parallel analysis supported two factors. Factor loadings ≥0.30 are shown in bold. EFA, exploratory factor analysis

SUPPLEMENTARY TABLE S2. Cross-tabulation of academic-integrity perceptions among LLMs users

**Perceived impact of LLMs use on academic integrity**	**Not concerned about potential academic misconduct**	**Concerned about potential academic misconduct**	**Total**
No perceived negative impact	95 (51.4%)	32 (17.3%)	127 (68.6%)
Perceived negative impact	2 (1.1%)	56 (30.3%)	58 (31.4%)
Total	97 (52.4%)	88 (47.6%)	185 (100%)

Note: Data are presented as n (% of all 185 LLMs users). The two questions assessed distinct aspects of academic-integrity perception and were therefore cross-tabulated and interpreted descriptively rather than treated as repeated measurements of the same binary outcome
